# Outcomes in Patients with Non-Small-Cell Lung Cancer with Brain Metastases: A Real-World Data Study from a Resource-Limited Country

**DOI:** 10.3390/cancers17101603

**Published:** 2025-05-09

**Authors:** Nevena Randjelovic, Marina Petronijevic, Marina Calamac, Marija Peulic, Biljana Filipovic, Vladan Mutavdzic, Aleksandar Djuric, Teodora Rankovic, Milos Bugarcic, Ivana Canak, Jelena Mikov, Nebojsa Igrutinovic, Stela Novak, Marko Marjanovic, Jelena Perovic, Teodora Urosevic, Tanja Cufer

**Affiliations:** 1 Department of Medical Oncology, University Clinical Centre of Kragujevac, 34000 Kragujevac, Serbia; marinakg2013@gmail.com (M.P.); marija.peulic@gmail.com (M.P.); biljanamarfil@hotmail.com (B.F.); vmutavdzic@yahoo.com (V.M.); teodora.rankovic.kg@gmail.com (T.R.); 2 Daily Hospital Care, Institute for Oncology and Radiology of Serbia, 11000 Belgrade, Serbia; marina.calamac@ncrc.ac.rs; 3 Oncology Institute of Vojvodina, 21204 Sremska Kamenica, Serbia; aleksandar.djuric@mf.uns.ac.rs (A.D.); novak.stela@onk.ns.ac.rs (S.N.); marjanovic.marko@onk.ns.ac.rs (M.M.); litavski.jelena@onk.ns.ac.rs (J.P.); urosevic.teodora@onk.ns.ac.rs (T.U.); 4 Institute for Pulmonary Diseases of Vojvodina, 21204 Sremska Kamenica, Serbia; milos.bugarcic@institut.rs (M.B.); ivana.canak@institut.rs (I.C.); jelenamikov33@yahoo.com (J.M.); 5 Department of Internal Medicine, Faculty of Medical Sciences, University of Kragujevac, 34000 Kragujevac, Serbia; shone31094@gmail.com; 6 Medical Faculty, University of Ljubljana, 1000 Ljubljana, Slovenia; tanja.cufer@mf.uni-lj.si

**Keywords:** lung cancer, brain metastases, NSCLC

## Abstract

Background: Real-world data provide insights into populations underrepresented in clinical trials such as non-small-cell cancer (NSCLC) patients with brain metastases (BMs). Despite global survival improvement due to novel drug employment, their impact in resource-limited settings like Serbia remains underexplored. This study analyzes the overall survival (OS) of NSCLC patients with BMs treated in routine clinical practice, considering patient-, disease- and treatment-related factors amid restricted access to novel drugs. Methods: We retrospectively analyzed 267 NSCLC patients diagnosed with BMs from 2018 to 2022 at a single Serbian clinical center. Inclusion required histologically confirmed NSCLC, radiologically verified BMs and complete clinical data. OS was defined as the time from BM verification to death or last follow-up. Kaplan–Meier curves and Cox regression were used for survival analysis. Results: Median OS (mOS) was 5.0 months. Univariate analysis linked age < 65 years, female gender, single BM, asymptomatic BMs, ECOG PS 0–1, BM verification at diagnosis and combined systemic and local therapy to better OS. Combined therapy offered the best survival rates (mOS: 9.0 months), while best supportive care and local-only therapy both resulted in a poor mOS of 2.0 months. Immunotherapy and targeted therapy were associated with the highest mOS, outperforming chemotherapy alone (13.0 vs. 7.0 months, *p* < 0.001). Multivariate analysis confirmed younger age, single BM, early BM verification and combined therapy as independent predictors of improved survival. Conclusions: limited access to novel therapies remains associated with poor patient survival, highlighting the need for better global availability.

## Simple Summary

This study addresses the lack of real-world data on life expectancy for metastatic lung cancer patients in Serbia, specifically focusing on NSCLC patients with brain metastases (BMs). Conducted retrospectively at a single academic center, 267 patients diagnosed with BMs from 2018 to 2022 were analyzed. The median overall survival rate (OS) was 5.0 months. Several factors linked to improved survival can affect a person’s outlook including age under 65, female gender, single and asymptomatic BM, good performance status (ECOG 0–1), BM verification at diagnosis and combined systemic and local therapy. Both immunotherapy and targeted therapy were associated with the longest survival (median OS: 13.0 months), while the poorest outcomes (median OS: 2.0 months) were recorded when using best supportive care and local-only therapy. This study highlights the survival gap in NSCLC patients with BMs without novel therapies, emphasizing the urgent need for improved global access to innovative treatments.

## 1. Introduction

Lung cancer (LC) remains one of the most prevalent cancers globally, with around 2.5 million new cases and the highest mortality, causing approximately 1.8 million deaths annually [1]. In Serbia, LC ranks first in incidence and mortality rates [2]. Non-small-cell lung cancer (NSCLC) comprises about 85% of all LC cases [3], with a 5-year survival rate for distant-stage disease below 10% [4]. Additionally, brain metastases (BMs) occur in 40–50% of patients with advanced NSCLC [5], particularly in those with epidermal growth factor receptor (EGFR) or anaplastic lymphoma kinase (ALK) oncogene mutations [6].

Sperduto et al. reported a median survival of 7.0 months for NSCLC patients with brain metastases (BMs) diagnosed between 1985 and 2005, influenced by factors such as histology, patients’ age, Karnofsky performance score, presence of extracranial metastases and number of BMs [7,8]. An updated analysis for patients treated from 2006 to 2014, which included EGFR and ALK status, showed improved survival up to 12 months overall with 15.2 months for adenocarcinoma and 9.2 months for non-adenocarcinoma patients [9]. This improvement was linked to the lack of testing in the non-adenocarcinoma cohort and targeted therapies, as EGFR-mutated patients naive to tyrosine kinase inhibitors (TKIs) at BM diagnosis had a longer survival rate; the median survival rate was 14 months for EGFR/ALK-negative patients, 23 months for EGFR-mutated and 45 months for ALK-positive patients [10].

Programmed death ligand 1 (PD-L1) also emerged as a prognostic factor, with lung adenocarcinoma patients showing higher PD-L1 expression and experiencing an improved median survival rate. As a result, PD-L1, along with EGFR and ALK status, was incorporated into the updated Graded Prognostic Assessment (GPA) score [11].

Survival in NSCLC patients with BMs can be significantly extended, especially in cases of targetable metastatic NSCLC (mNSCLC). EGFR mutations enhance sensitivity to radiation, with preclinical data showing increased cytotoxicity when combined with EGFR-TKIs. The third generation of EGFR-TKIs offers strong CNS penetration and efficacy while CNS-active ALK-TKIs show similar benefits for ALK-positive patients [12]. Osimertinib, a third-generation EGFR-TKI, demonstrated high intracranial efficacy in the FLAURA trial with 20 of 22 EGFR-mutant patients with evaluable BM achieving an intracranial response [13]. Likewise, lorlatinib, a third-generation ALK-TKI showed an 82% intracranial response rate in treatment-naive ALK-positive NSCLC patients in the CROWN trial [14].

In the context of immunotherapy, a pooled analysis of PD-L1-positive advanced or mNSCLC patients from the KEYNOTE-001, -010, -024, and -042 trials showed that pembrolizumab provided similar progression-free survival benefits in patients with and without baseline BMs. However, BMs remained a negative prognostic factor as overall survival (OS) was lower in patients with baseline BMs compared to those without them [15]. The ATEZO-BRAIN study demonstrated that atezolizumab combined with chemotherapy achieved intracranial responses comparable to systemic responses, potentially delaying the need for local therapy and helping maintain quality of life [16].

Randomized controlled trials (RCTs), while essential for internal validity, often exclude broader patient populations, thus limiting their real-world applicability. Contrary to the above mentioned, real-world data (RWD) offer insights from more diverse clinical settings and help bridge gaps in evidence [17]. Sperduto et al. have recently reported a median overall survival (mOS) of 17 months for adenocarcinoma and 8 months for non-adenocarcinoma patients with BM [11]. In patients without targetable mutations, RWD from five European Union countries showed an mOS under one year ranging from 5 months in France to 9 in Germany, with a shorter survival rate seen in non-adenocarcinoma histology (5–6 months) compared to adenocarcinoma (9 months) [18]. Alarmingly, recent data indicate that mNSCLC patients with BMs not receiving systemic therapy may have an mOS as low as 2 months [19].

Unfortunately, there is a lack of data on the survival of metastatic lung cancer patients in Serbia based on RWD. Additionally, no specific studies have focused on NSCLC patients with BMs in this population. Therefore, this study aims to analyze the characteristics of NSCLC patients with BMs treated in routine clinical settings and to determine overall survival after BMs diagnoses considering patient-, disease- and treatment-related factors in the era before the introduction of novel targeted therapies in everyday clinical practice in our country.

## 2. Materials and Methods

We retrospectively collected NSCLC patients diagnosed with BMs at any point during their disease course between 1 January 2018 and 31 December 2022. Data were collected from electronic archives at a single academic clinical center in Serbia.

**Figure 1:**
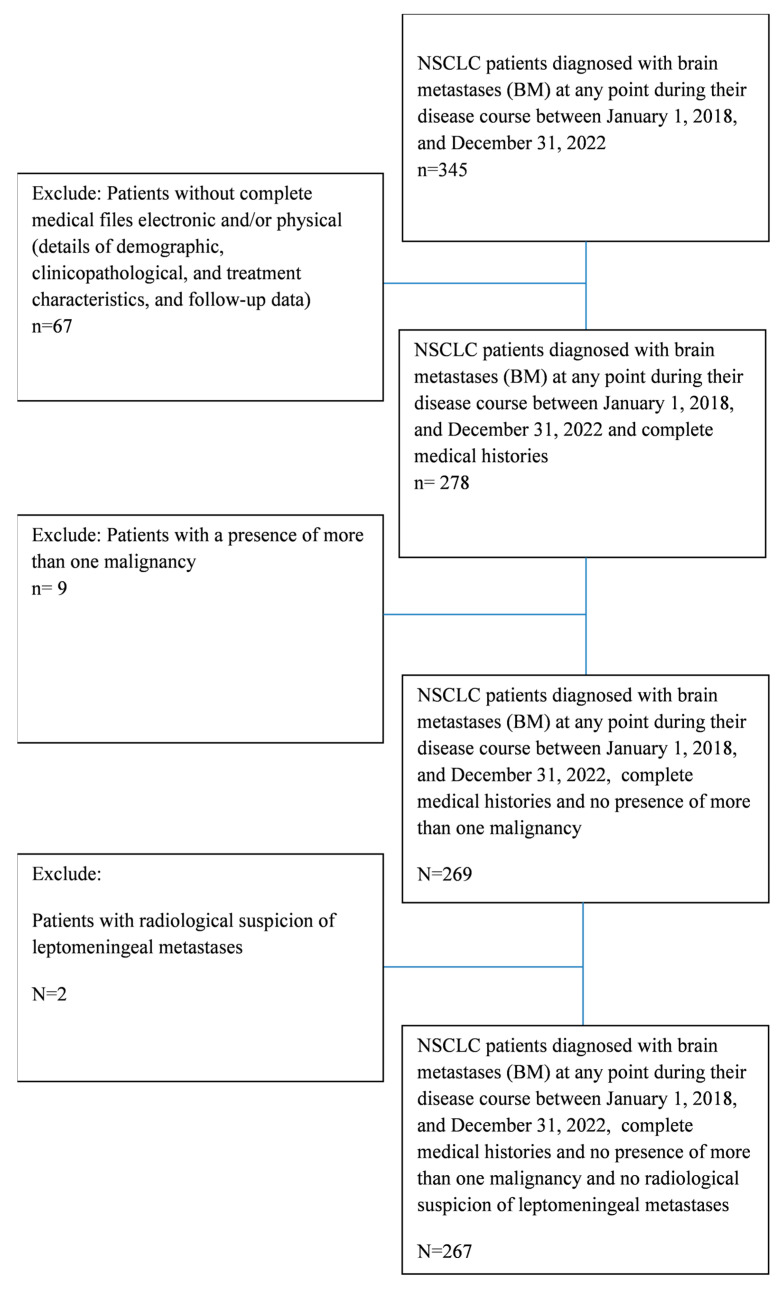
Patient collection flow chart.

The inclusion criteria required histologically confirmed NSCLC, detection of BMs through magnetic resonance imaging (MRI) or computed tomography (CT) at any stage of the disease or treatment from the time of NSCLC diagnosis until death or last follow-up. Additionally, complete clinical data and follow-up information had to be available. Patients without complete electronic and/or physical medical files were excluded, as well as those with the presence of more than one malignancy or radiological suspicion of leptomeningeal metastases (Figure 1). Patient follow-up was finished on the date of 25 May 2024, thus ensuring a minimum 2-year follow-up for all patients included.

We extracted socio-demographic patient data, diagnosis details (radiological and pathological), treatment and follow-up information and Eastern Cooperative Oncology Group performance status (ECOG PS) data, which allowed us to track patients from diagnosis to the end of treatment, death or last follow-up. Overall survival was evaluated as the duration of time from the BM radiological verification until death or the last follow-up. Patients’ personal data were completely anonymized.

In Serbia, oncology care is delivered within a universal healthcare system, with patients receiving treatment through mandatory national health insurance. The timing of cranial radiological evaluation was determined by the treating clinician. In the studied group, the type of NSCLC was identified using standard immunohistochemistry (IHC) on biopsy or surgical tissue samples. During the period covered by this study, molecular testing was limited. Since 2020, PD-L1 IHC testing has been available on demand for all mNSCLC patients with an ECOG performance status (PS) of 0–1. EGFR polymerase chain reaction (available throughout the study period) and ALK IHC testing (available since 2020) were performed on demand for patients with unresectable adenocarcinoma, adenosquamous carcinoma, or not-otherwise-specified (NOS) histology. For squamous cell carcinoma, these tests were limited to younger, non-smoking patients. EGFR testing was available for patients with ECOG PS 0–1, while ALK testing was permitted for those with ECOG PS 0–2 if brain metastases (BMs) were the cause of the PS decline.

Molecular testing referrals reflected both treatment availability and reimbursement policies. Novel therapies are approved by the national health insurance fund based on indications established by RCTs and eligibility criteria, as specified in national reimbursement guidelines. As a result, patients with poorer PS were not evaluated for actionable mutations but would be reassessed for testing and treatment if their PS improved. Patient treatment followed national health insurance guidelines, which included access to targeted therapy and immunotherapy—available predominantly in the first-line setting for advanced unresectable or metastatic NSCLC, mostly since 2020. More specifically, during the study period, first- and second-generation EGFR-TKIs were available for first-line use, while osimertinib was restricted to second-line treatment for patients with a confirmed T790M mutation. Similarly, only second-generation ALK-TKIs were available for first-line therapy. Immune checkpoint inhibitors were available as first-line treatment exclusively for patients with PD-L1 expression ≥ 50%.

### Statistical Analysis

The commercial, standard software package SPSS version 20.0 was used for statistical data processing. (The Statistical Package for Social Sciences software (IBM SPSS Statistics for Windows, Version 20.0. Armonk, NY, USA: IBM Corp.)). Each of the prognostic variables was evaluated using the long rank method, after which the significant variables were entered into the Cox hazards univariate and multivariate regression model for the assessment of predictive significance. Prognostic overall survival was analyzed using Kaplan–Meier curves. Values of *p* < 0.05 were considered statistically significant.

## 3. Results

The majority of our sample consisted of male patients (60.7%) of age <65 years (60.3%) and of non-squamous NSCLC histology (81.6%). Detailed samples’ characteristics are presented in Table 1. Representation of molecular testing and applied systemic treatment is shown in Table 2. In our study, 165 patients (61.8%) underwent a CT scan of the brain to assess intracranial disease spread at initial diagnosis and/or at the moment of symptom appearance indicating intracranial disease, while 101 patients (37.8%) had an MRI, and only one patient (0.4%) underwent both.

**Figure 2:**
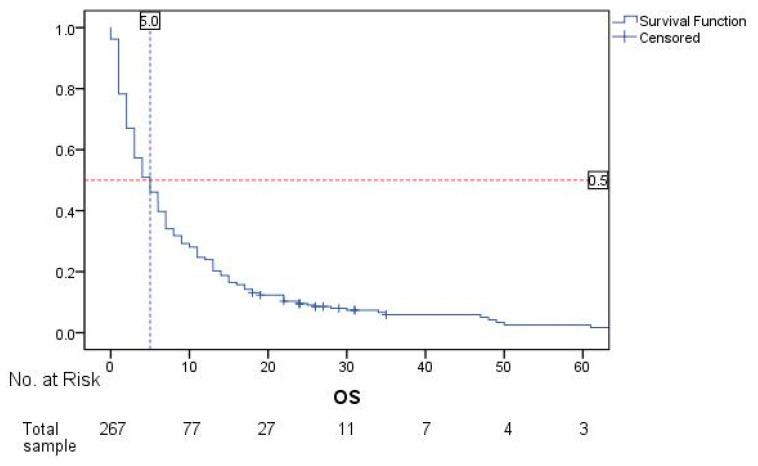
Overall survival rates of all patients (n = 267) from the moment of BM diagnosis.

For the total sample, calculated median OS in months is 5.0 (CI 95%, 3.981–6.019) (Figure 2). Median follow-up time for OS in months is 35.0 (CI 95% 19.064–50.936).

Univariate analysis showed that statistically significant factors influencing the OS in the studied population were age, gender, ECOG PS, timing of BM verification, number of BMs, symptoms and BM treatment (Table 3). Significantly better survival was recorded in patients with age < 65 years (5.0 vs. 3.0 months, *p* = 0.029), female patients (6.0 vs. 4.0 months, *p* = 0.038), patients with ECOG PS 0–1 (9.0 vs. 3.0 months, *p* < 0.001), patients who were diagnosed with BMs earlier in the disease—at first diagnosis and at later stage IV diagnosis (6.0 and 4.0 vs. 2.0 months, *p* = 0.002), patients with only one brain metastasis (7.0 vs. 3.0 months, *p* < 0.001), patients with no symptomatic BMs (8.0 vs. 3.0 months, *p* < 0.001) and patients treated with combined systemic + local treatment, with mOS being 9.0 months versus patients treated with best supportive care (BSC) and local-only treatment, both amounting to the mOS of only 2.0 months (*p* < 0.001).

**Figure 3:**
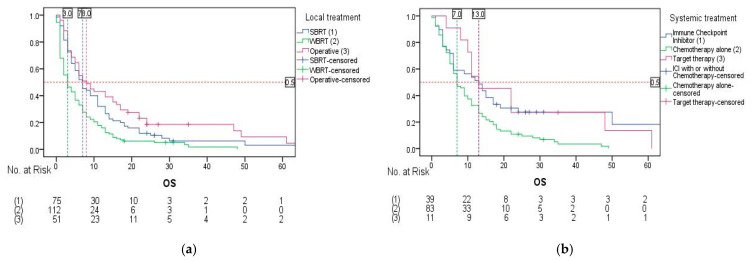
Overall survival rates by the type of local and systemic treatment: (**a**) overall survival rates by the type of local treatment; (**b**) overall survival rates by the type of systemic treatment.

The types of local and systemic treatments were excluded from the regression analysis evaluating the impact of these treatment modalities on OS due to the high proportion of untreated patients (29 patients (10.9%) did not receive any form of local treatment, and 134 patients (50.2%) did not receive any systemic treatment). Following the exclusion of patients with missing data, the log-rank test was performed. In regard to the type of local treatment, statistically significant differences in survival were found (*p* < 0.001). Patients treated with stereotactic body radiation therapy (SBRT) had an mOS of 7.0 months (95% CI: 4.695–9.305). Whole brain radiotherapy (WBRT)-treated patients yielded an mOS of 3.0 months (95% CI: 1.522–4.478), while operative treatment yielded an mOS of 8.0 months (95% CI: 4.819–11.181) (Figure 3a). In terms of systemic therapy, statistically significant differences in OS were also found (*p* = 0.003). Immune checkpoint inhibitor (ICI) treatment resulted in an mOS of 13.0 months (95% CI: 6.882–19.118). Chemotherapy alone yielded an mOS of 7.0 months (95% CI: 5.090–8.910), while targeted therapy demonstrated an mOS of 13.0 months (95% CI: 5.232–20.768) (Figure 3b).

**Figure 4:**
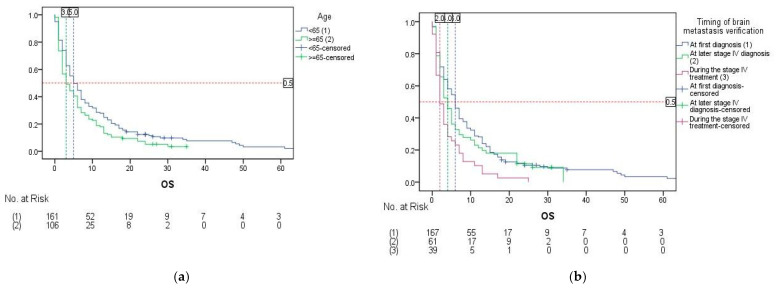

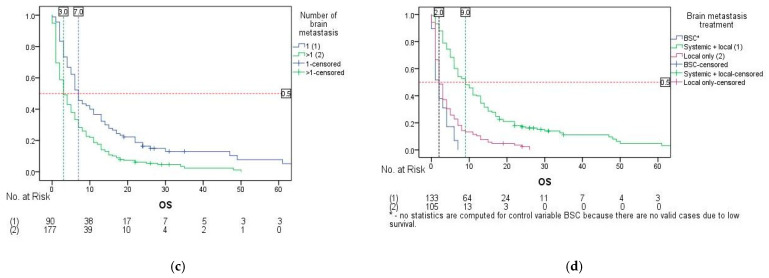
Overall survival rates by independent prognostic factors: (**a**) overall survival by age; (**b**) overall survival by the timing of the brain metastasis verification; (**c**) overall survival by the number of brain metastases; (**d**) overall survival by the type of brain metastasis treatment.

Multivariate Cox regression analysis included statistically significant variables from the univariate analysis as predictors—age, gender, ECOG PS, timing of BM verification, number of BMs, symptoms and BM treatment. An omnibus test showed that the overall model was significant (χ^2^ = 99.114, *p* < 0.001). Independent predictive factors of better OS with statistical significance were shown to be age < 65 years (Figure 4a), timing of BM verification at first diagnosis (*p* = 0.008) and at later stage IV diagnosis (*p* = 0.017) (Figure 4b), having only one BM (Figure 4c) and treatment of BMs with combined systemic + local treatment (*p* < 0.001) (Table 2). Only application of local treatment did not show statistical significance in the model (*p* = 0.180) (Figure 4d).

## 4. Discussion

Real-world data studies offer valuable insights that complement RCTs, particularly by evaluating current and potentially guiding the implementation of new treatment patterns. This is especially relevant for patients with brain metastases (BMs) who are frequently excluded from RCTs. Despite advances in targeted and immunotherapies that have reshaped the treatment landscape for metastatic mNSCLC, RWD continues to highlight poor outcomes in this group. In populations primarily receiving palliative or local treatment for BMs, mOS remains low—reported as just 2 months in some studies [19]. Hatton et al. further showed that survival outcomes were better in patients with known driver mutations. For instance, EGFR-positive patients had a median survival of 4.8 months compared to 3.0 months for EGFR-negative and 1.8 months for those with unknown status. Similarly, patients with known ALK status—whether positive or negative—fared better than those with unknown expression (mOS 1.8 months) [19]. These findings align with Sperduto et al., who also found that unknown molecular status correlated with poorer survival [11]. These observations underscore the importance of early molecular testing and the integration of targeted therapies to improve outcomes. The REFLECT study also emphasized the need for reflex testing and early identification of actionable mutations at diagnosis to enable timely and effective treatment strategies [20].

The mOS in our cohort was 5.0 months, aligning with some published real-world data [18,19]. However, as OS was calculated from the time of BM confirmation, longer medians might be observed if measured from initial NSCLC diagnosis. Molecular testing in our study was performed selectively—primarily to guide first-line treatment with novel agents per local guidelines, which required ECOG PS 0–1. Consequently, a large proportion of patients were not tested—PD-L1 in 55.1%, EGFR in 43.4%, and ALK in 62.9% of cases. This on-demand testing strategy likely contributed to poor outcomes, as only 14.6% received pembrolizumab and 4.1% received EGFR-TKIs for BMs. This highlights that molecular testing was generally reserved for patients considered eligible for novel therapies based on restrictive guidelines. As a result, the selective testing and treatment approach likely influenced the observed mOS, inherently favoring those with better performance status and treatment potential.

We also observed differences in OS based on various clinical factors, providing further insight into our study outcomes. In univariate analysis, better OS was associated with age <65 years, female gender, the presence of a single brain metastasis, asymptomatic BMs, ECOG PS 0–1, earlier BM detection, and the use of combined systemic and local treatment. However, multivariate analysis identified only age, timing of BM diagnosis, number of BMs, and type of BM treatment as independent prognostic factors.

The impact of age on OS in patients with BMs is well established, and our findings are consistent with the existing literature. Sperduto et al. introduced the GPA in 2008, a prognostic index based on four factors: age, Karnofsky Performance Status, presence of extracranial metastases, and number of BMs [21]. Age remains a key prognostic factor in more recent models as well, such as the Lung-molGPA, where patients under 65 years continue to show improved survival outcomes in NSCLC with BMs [22].

In the original GPA model, Sperduto et al. did not identify patient gender as a significant prognostic factor [21]. However, Chen et al., similar to our findings, reported female gender as associated with better survival in univariate—but not multivariate—analysis [22]. More recent studies from 2023 identified male gender as an independent prognostic factor for worse survival [23,24], aligning with our results, although we observed this association only in univariate analysis. Overall, female gender is generally linked to more favorable outcomes in LC, potentially due to differences in histological subtypes, smoking habits and treatment patterns [24]. An Australian cohort study further supported this, showing that women—typically younger with fewer comorbidities and lower smoking rates—had improved survival rates [25]. Additionally, emerging evidence suggests that LC in women may have distinct pathological and immunological characteristics [26]. Further investigation is needed to better understand the biological and clinical drivers behind these gender-based survival differences, particularly in NSCLC patients with BMs.

Around 60% of our cohort had an ECOG PS ≥2, which may help explain the low median OS observed. However, since ECOG PS did not emerge as a significant factor in our multivariate analysis, this remains a hypothetical explanation. The prognostic value of performance status is well established, notably through Sperduto et al.’s work on the Lung-molGPA. Moreover, multiple studies—across both oncogene-addicted and non-oncogene-addicted NSCLC populations—have consistently demonstrated the strong influence of PS on survival outcomes [19,23,27].

In our study, the timing of BM verification emerged as an independent prognostic factor, with the most favorable outcomes observed in patients whose BMs were detected at the first NSCLC diagnosis. This may reflect limited treatment availability later in the disease course. Similarly, the REFLECT study reported an mOS of 21.8 months for patients with BMs at the start of first-line EGFR-TKI therapy compared to just 6.4 months for those who developed BMs during treatment—findings that are in line with our observations [20]. Although differences in patient populations and treatment strategies limit direct comparisons, these results underscore the need for further investigation.

Regarding the number of BMs, while Hatton et al. did not find this to be a significant prognostic factor [19], many other studies—including those by Sperduto et al. [7,8,9,10,11]—have demonstrated poorer survival outcomes in patients with multiple BMs across both oncogene-addicted and non-oncogene-addicted NSCLC cohorts [5,17,27,28,29]. Our results support this trend, although we categorized patients simply as having single vs. multiple BMs. A more nuanced stratification—based on the number or volume of BMs—could offer deeper insight into this relationship.

The absence of BM-related symptoms was associated with better survival in univariate analysis. This aligns with findings from Chen et al., who identified symptomatic BMs as an independent prognostic factor in patients with 1–4 NSCLC BMs [24], and with data from Steindl et al. showing similar results [30]. A larger sample size could help clarify this association in our study.

Both univariate and multivariate analyses showed improved OS in patients receiving combined systemic and local treatments. Commonly, patients were treated with chemotherapy as the systemic option and WBRT as the local option. Both univariate and multivariate analyses showed improved OS in patients receiving combined systemic and local treatments, most commonly chemotherapy with WBRT. While earlier randomized trials reported no OS benefit from adding WBRT to chemotherapy, they noted improved intracranial control at the cost of higher toxicity [31,32]. Similarly, the QUARTZ study found no OS benefit from WBRT, especially in patients over 70 [33]. The TURBO-NSCLC trial reported better local control—but not OS—with SBRT plus CNS-penetrant TKIs in EGFR- and ALK-driven NSCLC [34]. Nevertheless, recent meta-analyses show that combining EGFR-TKIs with radiotherapy significantly improves OS and the intracranial response [35,36], and a real-world Italian study found that upfront local treatment with TKIs or ICIs improved OS [37]. However, a recent multinational meta-analysis found no OS benefit when combining radiation with ICIs [38], contradicting our findings. These discrepancies may stem from methodological differences and treatment variations. Importantly, the lack of a subgroup treated with systemic therapy alone in our cohort limits definitive conclusions, highlighting the need for future research including such patients.

We excluded local and systemic treatment variables from Cox regression due to the small number of patients treated with targeted therapies and the large proportion receiving no local or systemic treatments. Despite this, log-rank analysis showed that SBRT and surgery were associated with better survival than WBRT, consistent with previous findings [39]. Systemic treatment comparisons were limited by disease pathology differences; ICIs were used for non-oncogene-addicted NSCLC, and targeted therapies were used for EGFR-mutated NSCLC. RCTs have highlighted the benefits of TKIs in EGFR-mutated NSCLC, with studies like LUX-Lung 3 and 6 showing improved PFS with afatinib compared to chemotherapy, even in patients with BMs [40]. The FLAURA trial further transformed treatment with osimertinib, achieving 91% intracranial response rates [13]. Real-world data also support osimertinib’s superior OS compared to earlier-generation TKIs [41]. However, limited availability of osimertinib in Serbia during the study period restricted its use. While in our study no patients received ALK inhibitors, previous trials such as CROWN have shown their intracranial efficacy [5,14]. Regarding ICIs, Hendriks et al. demonstrated comparable response rates in patients with and without BMs [15,42]. In our cohort, ICIs were available as first-line treatment only for patients with a PD-L1 score ≥50%. Broader access to both ICIs and EGFR-TKIs due to the introduction of novel indications since 2024 may open opportunities for further research and better understanding.

Our study had several limitations. Being retrospective, it introduced biases, including selection bias, due to the exclusion of patients with missing records or those lost to follow-up. The true number of NSCLC BMs at our institution could be higher, as routine cranial screening for asymptomatic patients is individualized. Small sample sizes in certain treatment subgroups prevented adequate comparison of systemic treatment options. Since this study was based on data from a single cancer center in Serbia, the findings may not fully reflect experiences across the country. The small number of patients with positive mutations limited further subgroup analysis. Additionally, factors like the presence of extracranial metastases and the timing of local treatment initiation were not included but could have influenced survival. These limitations can be addressed in future, expanded research.

## 5. Conclusions

This study highlights the poor survival of NSCLC patients with brain metastases in the absence of novel therapies, emphasizing the urgent need for broader global access to innovative treatments. It reveals significant disparities in treatment availability and underscores the transformative potential of new therapies.

Taking into consideration the fact that we conducted the first study of this specific patient population in Serbia, we believe that a future multi-institutional study, which is supposed to include more patients treated with novel therapies, could offer valuable insights into a wider array of patient-, disease- and treatment-related factors affecting survival rates. In resource-limited settings like Serbia, optimizing care for brain metastases requires the timely involvement of multidisciplinary teams to guide individualized treatment decisions by adapting local treatment options when systemic therapies are limited. Mandatory cranial imaging is essential to ensure the prompt and appropriate management of brain metastases. Furthermore, adapting guidelines to include reflex molecular testing, increasing participation in international clinical trials and advocating for evidence-based policy changes can improve access to advanced therapies and enhance patient outcomes. Although treatment availability in Serbia is gradually improving, challenges still exist. Ongoing real-world data collection not only supports further research but also holds the potential to improve clinical practice and patient outcomes by identifying populations at greater risk.

## Figures and Tables

**Table 1:** Demographic, clinicopathological, and treatment characteristics of the study population.

Variable		Patients	
		Number	Percentage
Age	<65	161	60.3
	≥65	106	39.7
Gender	Male	162	60.7
	Female	105	39.3
Smoking status	Smokers and ex-smokers	197	73.8
	Non-smokers	70	26.2
Histologic type	Squamous cell lung cancer	49	18.4
	Non-squamous cell lung cancer	218	81.6
ECOG performance status	PS 0–1	110	41.2
	PS ≥ 2	157	58.8
Stage IV	At first diagnosis	196	73.4
	During the disease course	71	26.6
Timing of brain metastases verification	At first diagnosis	167	62.5
	At later stage IV diagnosis	61	22.8
	During the stage IV treatment	39	14.6
Number of brain metastases	1	90	33.7
	>1	177	66.3
Symptoms	Yes	145	54.3
	No	122	45.7
Brain metastases treatment	BSC	29	10.9
	Systemic + local	133	49.4
	Local treatment only	105	39.3
Local treatment ^1^	SBRT	75	28.1
	WBRT	112	41.6
	Operative	51	19.1
Systemic treatment ^2^	Immune Checkpoint Inhibitor	39	14.6
	Chemotherapy alone	83	31.1
	Target therapy	11	4.1

ECOG—Eastern Cooperative Oncology Group; BSC—best supportive care; SBRT—stereotactic body radiation therapy; WBRT—whole brain radiotherapy. ^1^ Twenty-nine (10.9%) patients were not treated with any local treatment. ^2^ One hundred and thirty-four (50.2%) patients were not treated with any systemic treatment.

**Table 2:** Data on molecular testing and applied systemic treatment.

Molecular Test	Test Results	Patients	Systemic Treatment		
			Immune Checkpoint Inhibitor	Chemotherapy Alone	Target Therapy
PD-L1	≥50%	39 (14.6%)	39 (29.4%)	0	0
	1–49%	24 (9.0%)	0	12 (9.0%)	3 (2.3%)
	<1%	57 (21.3%)	0	28 (21.1%)	2 (1.5%)
	Not tested	147 (55.1%)	0	43 (32.3%)	6 (4.5%)
EGFR	Wild-type	132 (49.4%)	30 (22.6%)	54 (40.6%)	0
	Mutated	19 (7.1%)	0	4 (3.0%)	11 (8.3%)
	Not tested	116 (43.4%)	9 (6.8%)	25 (18.8%)	0
ALK	Positive	0	0	0	0
	Negative	99 (37.1%)	28 (21.1%)	37 (27.8%)	5 (3.8%)
	Not tested	168 (62.9%)	9 (6.8%)	83 (62.4%)	0

ALK—anaplastic lymphoma kinase; EGFR—epidermal growth factor receptor; PD-L1—programmed death ligand 1.

**Table 3:** Univariate and multivariate analysis of overall survival.

Variable			Univariate Analysis		Multivariate Analysis	
		mOS in Months (95% CI)	HR (95% CI)	*p*	HR (95% CI)	*p*
Age	<65	5.0 (3.537–6.463)	0.752 (0.583–0.971)	**0.029**	0.760 (0.580–0.994)	**0.045**
	≥65	3.0 (1.448–4.552)			1	
Gender	Male	4.0 (3.066–4.934)	0.762 (0.590–0.985)	**0.038**	1	0.135
	Female	6.0 (4.661–7.339)			0.815 (0.623–1.066)	
Smoking status	Smokers and ex-smokers	5.0 (3.791–6.209)	0.859 (0.651–1.134)	0.285	-	-
	Non-smokers	4.0 (2.277–5.723)			-	
Histologic type	Squamous cell lung cancer	3.0 (1.753–4.247)	0.814 (0.592–1.120)	0.207	-	-
	Non-squamous cell lung cancer	6.0 (4.807–7.193)			-	
ECOG performance type	PS 0–1	9.0 (5.979–12.021)	0.478 (0.368–0.621)	**<0.001**	0.676 (0.418–1.093)	0.110
	PS ≥2	3.0 (2.060–3.940)			1	
Stage IV	At first diagnosis	5.0 (3.789–6.211)	0.917 (0.691–1.217)	0.548	-	-
	During the disease course	4.0 (2.826–5.174)			-	
Timing of brain metastases verification	At first diagnosis	6.0 (4.778–7.222)	0.533 (0.373–0.761)	**0.002**	0.610 (0.424–0.878)	**0.022 ^1^**
	At later stage IV diagnosis	4.0 (2.729–5.271)	0.614 (0.406–0.927)		0.594 (0.389–0.910)	
	During the stage IV treatment	2.0 (0.980–3.020)	1		1	
Number of brain metastases	1	7.0 (5.366–8.634)	0.569 (0.434–0.745)	**<0.001**	0.576 (0.432–0.769)	**<0.001**
	>1	3.0 (2.069–3.931)			1	
Symptoms	Yes	3.0 (2.351–3.649)	0.511 (0.396–0.659)	**<0.001**	1	0.428
	No	8.0 (5.540–10.460)			1.218 (0.748–1.983)	
Brain metastases treatment	BSC	2.0 (1.317–2.683)	1	**<0.001**	1	**<0.001 ^1^**
	Systemic + local	9.0 (6.828–11.172)	0.237 (0.154–0.366)		0.320 (0.197–0.520)	
	Local treatment only	2.0 (1.352–2.648)	0.610 (0.402–0.928)		0.746 (0.486–1.145)	

ECOG—Eastern Cooperative Oncology Group; BSC—best supportive care; HR—hazard ratio; CI–confidence interval; mOS—median overall survival. ^1^ *p*-value for the whole variable. Bolded *p*-values indicate statistical significance (*p* < 0.05).

## Data Availability

The data presented in this study are available on request from the corresponding author due to the issue of maintaining the complete privacy of the participants.
